# Human testis-expressed (TEX) genes: a review focused on spermatogenesis and male fertility

**DOI:** 10.1186/s12610-021-00127-7

**Published:** 2021-04-22

**Authors:** Hela Bellil, Farah Ghieh, Emeline Hermel, Béatrice Mandon-Pepin, François Vialard

**Affiliations:** 1 Département de Génétique, CHI de Poissy St Germain en Laye, Poissy, France; 2 Université Paris-Saclay, UVSQ, INRAE, BREED, F-78350 Jouy-en-Josas, France; 3 Ecole Nationale Vétérinaire d’Alfort, BREED, F-94700 Maisons-Alfort, France

**Keywords:** Testis-expressed gene, TEX, infertilité masculine, spermatogenèse, modèle murin, défaut génétique, Testis-expressed gene, TEX, Male infertility, Spermatogenesis, Mouse model, Genetic defect

## Abstract

Spermatogenesis is a complex process regulated by a multitude of genes. The identification and characterization of male-germ-cell-specific genes is crucial to understanding the mechanisms through which the cells develop. The term “*TEX* gene” was coined by Wang et al. (Nat Genet. 2001; 27: 422–6) after they used cDNA suppression subtractive hybridization (SSH) to identify new transcripts that were present only in purified mouse spermatogonia. *TEX* (*Testis expressed*) orthologues have been found in other vertebrates (mammals, birds, and reptiles), invertebrates, and yeasts. To date, 69 *TEX* genes have been described in different species and different tissues. To evaluate the expression of each *TEX/tex* gene, we compiled data from 7 different RNA-Seq mRNA databases in humans, and 4 in the mouse according to the expression atlas database.

Various studies have highlighted a role for many of these genes in spermatogenesis. Here, we review current knowledge on the *TEX* genes and their roles in spermatogenesis and fertilization in humans and, comparatively, in other species (notably the mouse). As expected, *TEX* genes appear to have a major role in reproduction in general and in spermatogenesis in humans but also in all mammals such as the mouse. Most of them are expressed specifically or predominantly in the testis. As most of the *TEX* genes are highly conserved in mammals, defects in the male (gene mutations in humans and gene-null mice) lead to infertility. In the future, cumulative data on the human *TEX* genes’ physiological functions and pathophysiological dysfunctions should become available and is likely to confirm the essential role of this family in the reproductive process. Thirteen *TEX* genes are now referenced in the OMIM database, and 3 have been linked to a specific phenotype. *TEX11* (on Xq13.1) is currently the gene most frequently reported as being associated with azoospermia.

## Résumé

La spermatogenèse est un processus complexe régulé par une multitude de gènes. L’identification et la caractérisation des gènes spécifiques des cellules germinales mâles sont essentielles pour comprendre les mécanismes par lesquels les cellules se développent. Le terme «gène *TEX*» a été inventé par Wang et al. (Nat Genet. 2001; 27: 422–6) après avoir utilisé l’hybridation soustractive d’ADNc (SSH) pour identifier de nouveaux transcrits qui n’étaient présents que dans la spermatogonie de souris. Puis, des orthologues *TEX* ont été trouvés chez d’autres vertébrés (mammifères, oiseaux et reptiles), des invertébrés et des levures. À ce jour, 69 gènes *TEX* (*Testis expressed*) ont été décrits dans différentes espèces et différents tissus. Pour évaluer l’expression de chaque gène *TEX/tex*, nous avons compilé les données de 7 bases de données différentes d’ARNm RNA-Seq chez l’homme, et 4 chez la souris selon la base de données de l’atlas d’expression.

Diverses études ont mis en évidence le rôle de plusieurs de ces gènes dans la spermatogenèse. Ici, nous passons en revue les connaissances actuelles sur les gènes *TEX* et leurs rôles dans la spermatogenèse et la fécondation chez l’humain et, comparativement, chez d’autres espèces (notamment la souris). Comme prévu, les gènes *TEX* semblent avoir un rôle majeur dans la reproduction en général et dans la spermatogenèse chez l’homme, mais aussi chez d’autres mammifères comme la souris. La plupart d’entre eux sont exprimés spécifiquement ou principalement dans les testicules. Comme la plupart des gènes *TEX* sont hautement conservés chez les mammifères, des défauts chez le mâle (mutations géniques chez l’homme et KO murin) conduisent à l’infertilité. À l’avenir, l’accumulation des données sur les fonctions physiologiques et les dysfonctionnements physiopathologiques des gènes *TEX* humains devraient devenir disponibles et confirmer le rôle essentiel de cette famille dans le processus de reproduction. Treize gènes *TEX* sont désormais référencés dans la base de données OMIM, et 3 ont été liés à un phénotype spécifique. *TEX11* (sur Xq13.1) est. actuellement le gène le plus fréquemment rapporté comme étant associé à l’azoospermie.

## Introduction

Male and/or female infertility (defined as the inability to conceive a child within 1 year of regular unprotected intercourse) affects up to 15% of couples [1]. Infertility is due to male factors in 40–50% of couples and can be due to environmental exposure, infections, immune problems or hormone deficiencies [2]. In 15–30% of all cases, genetics factors are involved [3].

Male germ cell development (spermatogenesis) is a tightly regulated developmental process that occurs through successive mitotic, meiotic and post-meiotic phases (in spermatogonia, spermatocytes and spermatids, respectively) [4]. During spermatogenesis, gene expression is regulated in three ways: intrinsically, interactively and extrinsically. The intrinsic program determines which genes are used and when these genes are expressed. The interactive regulation involves communication between germ cells and somatic cells. Lastly, the extrinsic program influences the interactive process through hormonal regulation [5].

The regulation of spermatogenesis involves the expression of a large number of genes in a precise cell- and stage-specific program [5]. A comprehensive understanding of spermatogenesis requires the identification and functional characterization of the 2300 or so genes that are predominantly expressed in the testes [6]. In the 2000s, the use of cDNA (complementary DNA) library construction techniques and the comparison of gene transcription profiles under different physiological conditions enabled the identified of genes specifically expressed in testis or gonads (named as the testis-expressed *(Tex*) genes). However, no information on the new gene family’s function (notably in the testis) was initially available [7].

Here, we review current knowledge on the *TEX* genes in humans and other species (notably the mouse) and focus on the genes’ roles in spermatogenesis and fertilization. Importantly, some of the *TEX* genes constitute promising biomarkers of male infertility.

## How the first *TEX* genes were identified and named

The term “TEX” for testis-expressed was coined by Wang et al. after they used cDNA suppression subtractive hybridization (SSH) to identify new transcripts that were present only in purified mouse spermatogonia [7]. Ten of the 23-novel germ-cell-specific genes, highly or exclusively testis-expressed (*Tex11* to 20) had not been annotated previously, and the human *TEX* orthologs were subsequently described [7]. Most of these genes have since been found to have a function in spermatogenesis, and additional *TEX* genes have been identified.

Before Wang et al.’s report, the *Tex* genes had been confused with the t-complex testis-expressed (*Tctex*) genes. The mouse t-complex corresponds to a portion of mouse chromosome 17 that had been identified in mouse t-haplotypes [8]. This t-haplotype contains four non-overlapping, paracentric inversions that span a genetic distance of 20 cM (centiMorgan). This results in a 100–200-fold suppression of recombination, which in turn keeps the haplotypes intact and leads to their divergence from the wild-type chromosomes. There are relatively few *Tctex* genes: *Tctex3, Tctex7, Tctex8, Tctex9, Tctex10, Tctex11*, and *Tctex12*. The genes are expressed predominantly in the germ cells of the testis, and map to various regions of the t-complex. Three genes are more abundantly expressed at the pachytene stage; three others are expressed after meiosis, and one (*Tctex*10) is expressed at all stages of spermatogenesis [8]. Two orthologs have been observed in the human: *TCTEX6* (also named *TEX6*) and *PPP1R11* (*TCTEX5*). To date, 69 *TEX* or *Tex* genes have been described in humans or mouse models. However, as described below, these genes do not constitute a homogeneous family; in contrast as the HOX (homeobox) or PAX (paired box) or RHOX (X-linked reproductive homeobox) genes with high sequence identity and very similar functions, the sole common feature of the Tex/TEX genes is their expression (solely or primarily) in the testis. After Wang et al.’s report, new testis-specific genes have been included in the TEX family and numbered sequentially. The TCTEX and TEX gene families are not related as such.

## The *TEX* gene family

**Fig. 1:**
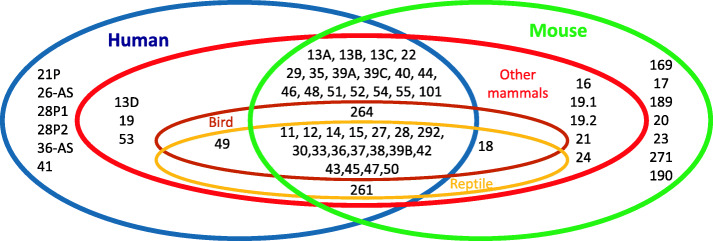
TEX expression according to species. *TEX* genes are common to the different species mentioned above, others are specific to each specie. Blue circle included *TEX* genes expressed in human, green circle included *Tex* genes expressed in mouse, red circle included *tex* genes expressed in mouse, red circle included *tex* genes expressed in other mammals, brown circle included *tex* genes expressed in birds, yellow circle included *tex* genes expressed in reptile

As mentioned above, 69 expressed *TEX* or *Tex* genes (61 human genes and 61 mouse genes (Fig. 1)) are listed in the main databases (https://gtexportal.org/home/, https://www.ensembl.org/index.html, https://www.omim.org/, etc.). These genes are distributed throughout the genome. To evaluate the expression of each *TEX/Tex* gene, we compiled data from 7 different RNA-Seq mRNA databases in humans, and 4 in the mouse according to the expression atlas database (https://www.ebi.ac.uk/gxa/home).

The 7 databases for RNA-Seq mRNA results in humans were:

1. the Genotype-Tissue Expression database (http://www.genome.ucsc.edu/gtex.html): 53 tissue.
2. Hallstrom et al.’s database [9]: 95 individuals representing 27 tissues.
3. the Uhlen laboratory’s database (https://www.proteinatlas.org/humanproteome): 122 individuals representing 32 tissues.
4. the Illumina Body Map [10]: 16 tissues.
5. the ENCODE project database from Snyder’s lab (https://www.encodeproject.org/): 13 tissues.
6. the mammalian database from Kaessmann’s lab [11]: 6 tissues, used to investigate the evolution of gene expression levels in different organs.
7. the Functional Annotation of the Mammalian Genome (FANTOM) 5 project (https://fantom.gsc.riken.jp/data/): 57 tissues

The 4 databases for RNA-Seq mRNA results in the mouse were:

1. the mammalian database from Kaessmann’s lab [11]: 6 tissues (as in humans).
2. the FANTOM database 5 projects: (https://fantom.gsc.riken.jp/data/): 35 tissues
3. the strand-specific RNA-seq of nine C57BL6 mouse tissues: 8 tissues.
4. Soumillon et al.’s database on brain, liver, and the whole testis [12]: 3 tissues

The data are reported in Table 1 (for humans), Table 2 (for mice), and Table 3 (for other species). For each gene, the highest level of tissue mRNA expression and the mean testis ratio (the ratio between testis expression and the total expression level for all other tissues) were reported in humans and mice. Protein expression evaluated according to the Human Protein Atlas (https://www.proteinatlas.org/) and the Human Proteome Map [13] was only reported for humans. The site of expression in the human testis was reported according to the Human Protein Atlas.

**Table 1:**
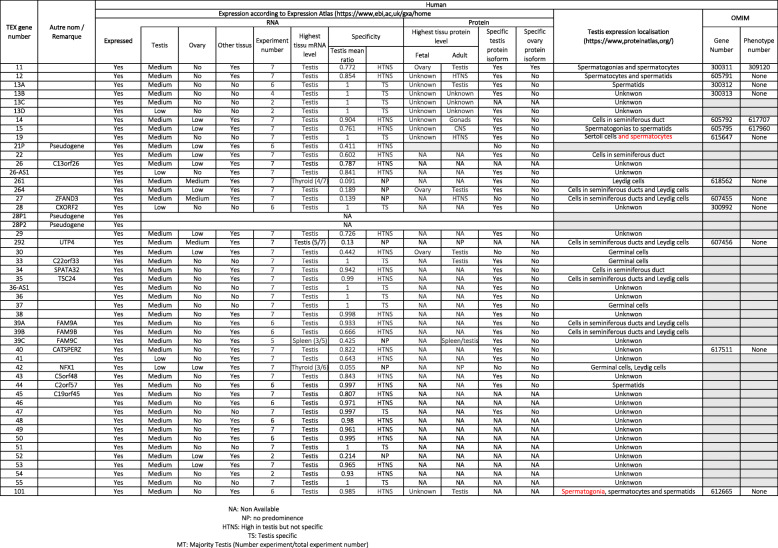
Human mRNA *TEX* gene expression, TEX expression and localization, OMIM reference

**Table 2:**
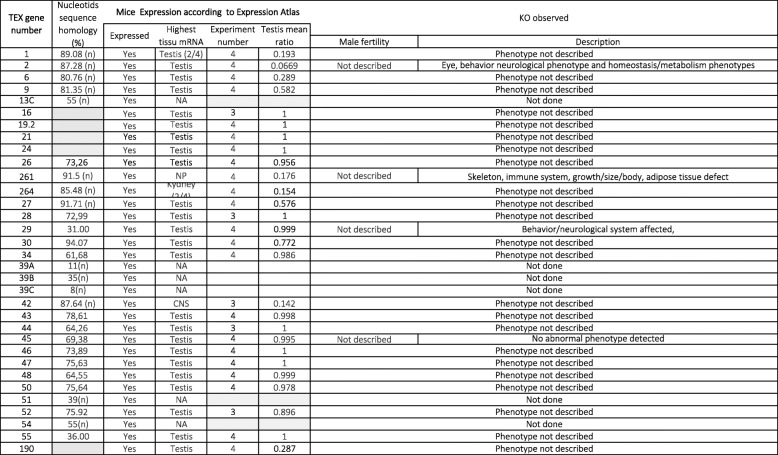
Mouse mRNA *Tex* gene expression, Nucleotids sequence homology (%) and KO models

**Table 3:**
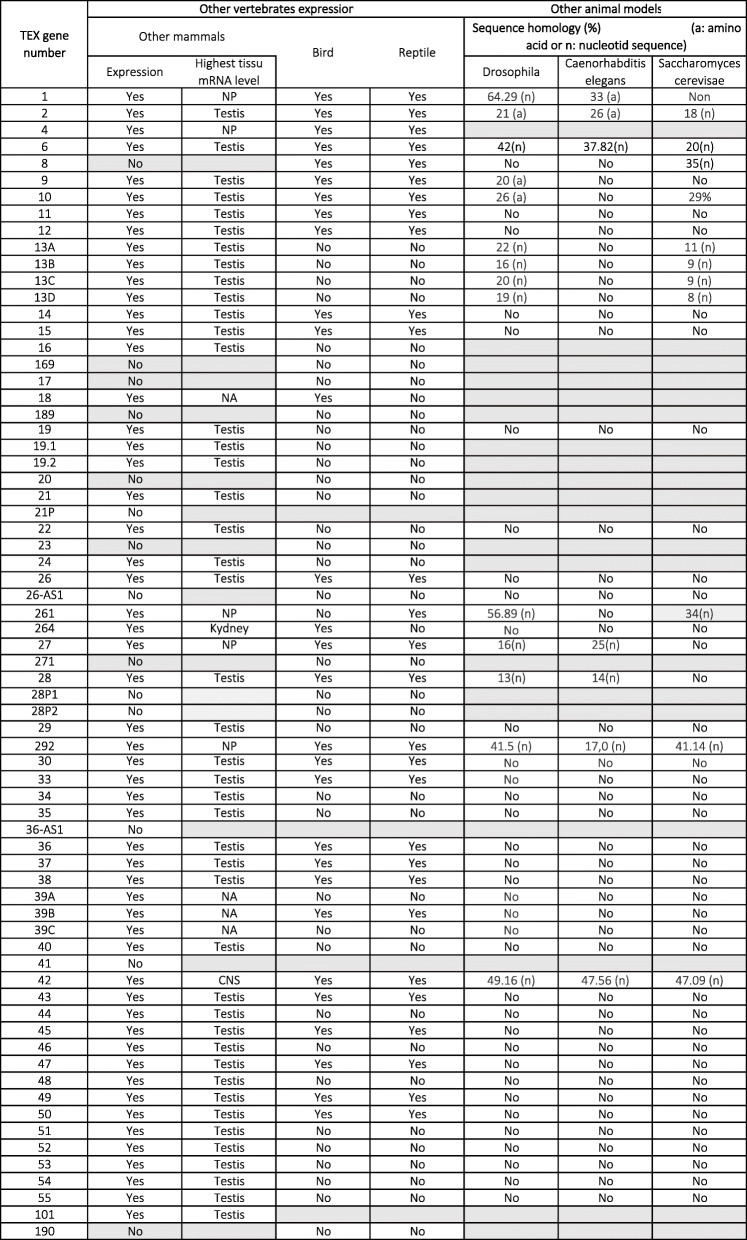
Others species mRNA *tex* gene expression and sequence homology

*tex* orthologues have been found in other vertebrates (mammals (mainly *Rattus norvegicus*, *Macaca mulatta*, *Equus caballus* and *Bos taurus*), birds (*Gallus gallus*), and reptiles), invertebrates (*Drosophila melanogaster* and *Caenorhabditis elegans*), and yeasts (*Saccharomyces cerevisiae*) (Table 3). RNA expression in species other than the human was reported according to the Expression Atlas (https://www.ebi.ac.uk/gxa/home).

In the following paragraphs, *TEX1* to *TEX10* genes are not considered. As mentioned above, *TCTEX* genes are not considered to be members of the *TEX* family, even though mutations in some of them (such as *TEX8* and *CAPZA3*) result in male infertility in the mouse (due to sperm with abnormally shaped heads and poor motility)*.* Ultimately, only 62 *TEX* genes have been defined as such.

## Evolutionary conservation of the *TEX* genes

Data are summarized in Fig. 1

### Conservation of identity between vertebrates and invertebrates

Fifty-three of the 62 genes reported in Tables 1, 2, 3 do not have identified or reported orthologues in invertebrates. For the 9 other genes (all of which are testis-specific in humans), orthologues have been identified in *Drosophila melanogaster*, *Caenorhabditis elegans* and/or *Saccharomyces cerevisiae*. Five of the 9 genes (*TEX13A, B, C, D* and *TEX28*) are also testis-specific in other mammals – indicating a high degree of conservation and a strong probable impact on spermatogenesis. *TEX13* is thought to be the ancestral gene. However, a large proportion of the *TEX* genes appear to be vertebrate-specific - confirming the differences in spermatogenesis between vertebrates and invertebrates [14].

### Conservation of identity between vertebrates

Thirty-nine of the 62 genes reported in Tables 1, 2, 3 are expressed in humans, mice, and other mammals. Twenty are also expressed in birds and reptiles, 1 is only expressed in a bird, 1 is only expressed in a reptile, and 17 are not expressed in birds or reptiles (and so are probably mammal-specific). Thirty-three of the 39 genes are expressed specifically in the testis (in 10 cases) or predominantly in the testis (in 22 cases). For the remaining 6 genes, the highest level of gene expression is not observed in the testis. Four of these 6 genes are not even testis-specific (Table 1).

Of the 62 genes reported in Tables 1, 2, 3, 6 are solely expressed in human. Three are pseudogenes (*TEX21P*, *28P1* and *28P2*), 2 considered to code for antisense RNAs (*TEX26-AS1* and *TEX36-AS1*), and the sixth is *TEX41.* The RNA expression data (when available) suggests that these 6 genes are testis-specific or at least much more strongly expressed in the testis than in other tissues. Seven of the 62 genes reported in Tables 1, 2, 3 are only expressed in mice; 3 of these are testis-specific expression or a very high testis expression level. For the last 10 genes, RNA expression has been identified in other vertebrates (but not in humans or mice) and appears to be generally testis-specific (mean testis ratios: from 0.944 to 1).

### Conservation of identity between humans and mice

When comparing humans and mice, the degree of nucleotide identity sequence ranges from 8.00% (for *TEX39C*) to 94.07% (for *TEX30*). However, when considering only those genes (*n* = 26) with a mean testis RNA ratio below 0.5 in humans and mice, the degree of nucleotide sequence identity ranges from 31.0% (for *TEX29*) to 86.45% (for *TEX12*). The 8 genes that are testis-specific in humans are also testis-specific in the mouse. Given the high observed degree of identity, studies of *TEX* gene function in the mouse are likely to be relevant.

## *Tex* gene expression and function in the mouse

*Tex* gene was first reported by Wang et al. in 2001 [7]. Ten of the 25 testis expressed genes, 10 (*Tex11* to *20)* had not been described previously, and 3 (*Tex15*, *19* and *20*). were expressed in the ovary. A human ortholog has been identified in 5 cases (*Tex*11 to 15). The analysis of testis cDNA libraries has since enabled the discovery of many other similar genes. Of the 52 *Tex* genes expressed in the mouse, 13 are testis-specific and 37 have a mean testis ratio below 0.5. For the 8 *Tex* genes with a mean testis ratio above 0.5, the testis is the tissue with the highest RNA expression level. The remaining genes have not been characterized. The available data are summarized in Table 4 and detailed in Table 2.

**Table 4:** Mouse *Tex* gene expression in the testis

	Highest expression in the testis					Highest expression in other tissues
Mean testis ratio	1	1 to 0.8	0.8 to 0.6	0.6 to 0.4	below 0.4	
*Tex* genes	13A, 13B, 16, 17, 19.2, 21, 22, 24, 28, 44, 46, 47, 55	11, 12, 14, 15, 19.1, 26, 29, 33, 34, 35, 36, 37, 38, 40, 43, 45, 48, 50, 52, 101	189, 20, 30	27	190	169, 23, 261, 264, 271, 292, 42

One or more mouse models (mainly knock-out models) have been produced for 47 of the 52 *Tex* genes. These models have been used to study the *Tex* genes’ function and the resulting level of male fertility. In fact, the male fertility phenotype is not known or has not been reported for 25 genes. For *Tex20, 23, 169, 189* and *292*, the mouse model is embryo-lethal. For the other genes, transgenic male mice are fertile in the *Tex22, 33, 35, 36, 37* and *271* models, subfertile in the *Tex 17, 18* and *40* models, and infertile in the *Tex11, 12, 14, 15, 19, 19.1, 38* and *101* models. Below, we provide detailed functional information for a small number of these *Tex* genes.

### Tex11

The *Tex11* gene codes for a protein with a tetratricopeptide repeat motif (mediating protein-protein interactions) and a meiosis-specific domain Spo22 [27, 28]. The gene has 175 orthologs, and the human vs. mouse sequence identify is 74%. In the mouse, TEX11 protein is observed in the cytoplasm and nuclei of type B spermatogonia, with the highest level in zygotene spermatocytes and a basal level in late pachytene spermatocytes [29]. It is the first X-encoded meiosis-specific factor to have been identified in the mouse. The abundant expression of TEX11 protein in type B spermatogonia and early spermatocytes suggest that *Tex11* has a key role in the early stages of germ cell development. The generation of *Tex11*-deficient mice has enabled researchers to elucidate the encoded protein’s role in spermatogenesis. In 2008, Yang et al. generated a *Tex11*-null mice by deleting 27 of the gene’s 30 exons. Consequently, spermatogenesis was impaired due to chromosomal asynapsis at the pachytene stage and a low level of crossover formation at the anaphase I stage. *Tex11*-deficient spermatocytes mostly undergo apoptosis at the pachytene stage, while surviving cells display chromosome nondisjunction at the first meiotic division - causes cell death and male infertility [30]. In another study, Adelman et al. generated a *Tex11* mutant strain by deleting gene exon 3, resulting in a frameshift and a termination codon. They found that the mutant males exhibited delayed repair of double-strand breaks (DSBs) in spermatocytes. DSB repair and chromosome synapsis are essential for genetic integrity; their dysfunction can cause various diseases, such as infertility [29]. *Tex11* is currently considered to be a constituent of the meiotic nodules involved in recombination and that interact with S*ycp2* (a component of the synaptonemal complex) [30].

### Tex12

*Tex12* is conserved among vertebrates. It encodes a 14.1 kDa meiosis-specific protein that does not contain any known protein domains. *Tex12* is specifically located in the central element structure of the synaptonemal complex and is strongly expressed in spermatocytes and spermatids during meiotic cell division exclusively [31, 32]. The gene codes for two transcripts (*Tex12–201* and *Tex12–201*) and has 116 orthologs. The human and mouse deduced open reading frames code for a 123-residue protein with 86% identity. *Tex12*-null mice are infertile. Males show a failure of chromosomal synapsis, whereas females show the loss of ovarian follicles. It has further been demonstrated that *Tex12* is a member of the synaptonemal complex, which comprises eight proteins: SYCP1–3, SYCE1–3, tex12 and SIX6OS1 [33].

### Tex13

*Tex13* is an X-linked gene expressed exclusively in male germ cells. The *Tex13* family has 4 members. Wang et al. identified the first two human orthologs (*TEX13A* and *B*) in 2001 [7]. The degree of human vs. mouse nucleotide sequence identity for *Tex13* varies from one database to another. Lastly, 4 *Tex13* genes have been identified: according to Profile Alignment software (https://www.ibi.vu.nl/programs/pralinewww/), the percentage nucleotide sequence identity is 50% for *Tex13A*, 54% for *Tex13B*, 30% for *Tex13C*, and 32% for *Tex13D*. According to the UniProt database (www.uniprot.org/), the percentage is 23% for TEX13A, 31% for TEX13B, 6% for TEX13C, and 8% for TEX13D. The location of TEX13 proteins within germ cells is nuclear. Using a mouse testicular teratoma cell line (considered to possess the characteristics of male germ cells), Kwon et al. [34] demonstrated that TEX13 expression is regulated in a stage-specific manner at the translational level. The protein migrates first to the nuclei of spermatogenic cells and then to the redundant nuclear envelope of the spermatozoon during spermiogenesis. It is found in mature sperm [34]. Remarkably, TEX13 was found to possess transcriptional repressor activity; its overexpression in GC-2 cells altered the expression levels of 130 genes, suggesting that TEX13 might have a role in transcriptional regulation during spermatogenesis [34]. Lastly, *Tex13* was the first gene shown to be transcribed in spermatogonia and whose transcripts are then stored in a translationally inactive state until the late meiotic stage. Male mice hemizygous for a *Tex13a* or *Tex13b*-null allele exhibit normal fertility.

### Tex14

*Tex14* codes for a testis-specific protein. The open reading frame’s predicted 1450 amino acid sequence consists of an ankyrin repeat domain and a protein kinase-C domain. It shares 64% amino acid sequence identity with the predicted human TEX14 protein [35]. TEX14 is an essential component of male and female embryonic intercellular bridges. The protein is strongly expressed in the testis and, more specifically, in seminiferous duct cells (Sertoli cells, spermatogonia, spermatocytes, and spermatids) [35]. It is required for both the formation of intercellular bridges during meiosis and kinetochore-microtubule attachment during mitosis. TEX14 acts by promoting the conversion of midbodies into intercellular bridges [35]. *Tex14*-null adult male mice are sterile, while females are fertile [36]. *tex14*-null males lacked intercellular bridges that connect differentiating germ cells, and so spermatogenesis did not progress beyond the first meiotic division. TEX14 is essential for the maintenance of stable intercellular bridges in gametes of both sexes but their loss specifically impairs male meiosis. Although a low number of oocytes are present in *Tex14*-null neonatal ovaries, females are fertile [36].

### Tex15

*Tex15* codes for a serine-rich protein in the mouse and a 3176 amino acid protein in the human (sequence identity: 47%) [37]. *Tex15* gene is expressed in spermatogonia and early spermatocytes. Its expression is downregulated in pachytene spermatocytes and abundant in postmeiotic germ cells [25]. *Tex15*-null females are fertile, whereas males are sterile with a dramatically reduced testis size, and a complete lack of pachytene spermatocytes and postmeiotic germ cells [37]. During spermatogenesis, *Tex15* encodes for a testis-specific protein required for normal chromosome synapsis and meiotic recombination in germ cells. It is also necessary for DNA DSBs (double-strand breaks) repair. TEX15 might be functionally active at different stages in spermatogenesis. It has been postulated that TEX15 functions downstream of DSB repair by SPO11 (a subunit of a DNA topoisomerase VI-like protein complex that is essential for meiotic recombination) but upstream of DSB repair by RAD51 (RecombinaseA-like 51) and DMC1 (DNA meiotic recombinase 1) during the meiotic recombination [37]. It was recently reported that during spermatogenesis, TEX15 binds to MILI - a member of the P-element induced wimpy testis in *Drosophila* (PIWI) family and that is required for germ cell differentiation. TEX15 silences transposable elements that escape the first round of de novo genome methylation in male germ cells [38]. It has been postulated that TEX15 is an essential epigenetic regulator that might operate as a nuclear effector of MILI by silencing transposable elements through DNA methylation. It has also been reported that in fetal gonocytes, TEX15 interacts with MIWI2 (another PIWI family member) and is required for piwi-interacting-RNA-directed de novo DNA methylation of transposons [39].

### Tex18

*Tex18* is a small gene identified first in the mouse by Wang et al. It has a single 240 bp exon and is specifically expressed in male germ cells. The encoded protein does not have any identified domains. A human ortholog of *Tex18* has not yet been identified [40]. It was later confirmed that *Tex18* is expressed in spermatogonia and then in other stages of male germ cell development [40]. Male *Tex18*-null mice are subfertile because of abnormal sperm morphology and reduced motility - a phenotype known as asthenoteratozoospermia. Spermatid maturation is unsynchronized and partially impaired in the seminiferous tubules, suggesting that *Tex18* is predominantly expressed during spermatid differentiation.

### Tex19

Tex19 is a mammal-specific duplicate gene (since renamed Tex19.1 and Tex19.2) found in the mouse and rat. According to the UniProt database, tex19.1 expression in the embryo decreases after murine embryonic stem and germ cell differentiation. At later stages of development, Tex19 expression is limited to the germ line. tex19.1 transcripts have also been detected in mouse pluripotent stem cells. It is thought that tex19.1 encodes a protein expressed solely in germ cells and pluripotent cells. Male Tex19.1-null mice are infertile, with a defect in meiotic chromosome synapsis, the persistence of DNA DSBs during meiosis, and a loss of post-meiotic germ cells. It was further demonstrated that TEX19.1 [41] and its paralog TEX19.2 [42] interact with PIWI proteins in mouse adult testis to repress transposable genetic elements and maintain genomic stability through successive generations. Furthermore, TEX19.1 was shown to promote Spo11-dependent recombination in mouse spermatocytes [42]. Placental expression of Tex19.1 has also been observed [43]. Accordingly, Tex19.1-null mouse embryos exhibit intra-uterine growth retardation and have small placentas. The observation that mobilization of LINE-1 (Long interspersed nuclear element 1) retrotransposons is restricted by TEX19.1 in mouse embryonic stem cells [44] may explain the placental dysfunction and small size. Lastly, it was recently reported that TEX19.1 maintains acetylated SMC3 (Structural Maintenance of Chromosome 3) and sister chromatid cohesion in postnatal oocytes and prevents aneuploidy [45].

### Tex27 (Zfand3: zinc finger an1 domain-containing protein 3)

*Tex27* is exclusively expressed in adult mouse testis. It codes for a protein containing a zinc-finger domain in the carboxy terminal region and a transactivation domain in the amino terminal region. TEX27 may be a transcription factor that is preferentially expressed in postmeiotic cells during mouse spermatogenesis [46]. In bird and reptile models, it was reported that the gene codes for two different transcripts: a short form mainly expressed in the testis, and a long form in the ovary. Sequence analysis revealed an extra exon in the genomic structures of the avian and reptilian *ZFAND3* genes. TEX27’s physiological functions in the testis and ovary are thought to differ in terms of germ cell maturation and regulatory mechanisms [47]).

### Tex33

*Tex33* expression is testis-specific; the encoded protein is found in the cytoplasm of round spermatids but much less in elongated spermatids [48]. Given that spermatogenesis is normal in male *tex33*-null mice, Tex33 might not be essential [49].

### Tex36

*Tex36* expression is testis-specific but male *Tex36*-null mice are fertile with no observable defects in reproductive organs, suggesting that TEX36 is also dispensable to spermatogenesis [50].

### Tex37

Similarly to *Tex36*, male *Tex37* null mice are fertile and have no detectable defects (vs. wild-type mice) in the testis/body weight ratio, epididymal sperm count, and testicular and epididymal histology [51].

### Tex40

Tex40 protein (also referred to as CATSPERZ) is located in the principal piece of the flagellum. It may represent a late evolutionary adaptation that maximizes fertilization inside the female mammalian reproductive tract [52]. *Tex40*-null mice are fertile and have a normal sperm count and a normal sperm morphology. However, the flagellum is rigid – impairing motility and leading to reduced fertility in vivo and in vitro. The human CATSPERZ and murine *Catsperz* are both auxiliary subunits of sperm calcium channel pore-forming proteins involved in the activation of spermatozoon motility. It was recently suggested that downregulation of this protein is the cause of the low sperm motility observed in asthenozospermic males [53].

### Tex101

Tex101 is mainly expressed in testis (from spermatogonia to spermatids) but it also transcribed during oogenesis. Mouse TEX101 is a testicular-germ-cell-specific protein predominantly located on the plasma membrane of germ cells during gametogenesis. TEX101 is one of the 29 glycosylphosphatidylinositol-anchored proteins expressed in the mouse testis, where it might regulate ion channels through CATSPERZ (cation channel, sperm-associated, auxiliary subunit zeta). When spermatogenesis in the testis is complete, the TEX101 protein remains on the sperm surface - including the tail portion. TEX101 is then cleaved from the sperm surface and released into the seminal fluid and is no longer detectable in male germ cells. The protein interacts with various molecules during the post-testicular maturation of spermatozoa, including some members of a disintegrin and metalloproteinase (ADAM) family [54]. In humans, TEX101’s role and interactome have yet to be determined.

Although Tex101-null mice produce spermatozoa and oocytes with a normal morphology, males are infertile. Sperm physiology and motility are abnormal, which impair sperm migration into the oviduct and hinder the acrosome reaction. TEX101 is therefore essential for male fertility; it has been suggested that TEX101 operates as a cell surface chaperone involved in the maturation of proteins required for sperm migration and sperm-oocyte interaction (such as Adam3) [49–52, 54–57].

### Tex261

*Tex261* is highly expressed in adult testis in general and in the Sertoli cells in particular. It is first expressed after 15 days of post-natal life, which coincides with the presence of pachytene cells from the first wave of meiosis during spermatogenesis. *TEX261* expression is not restricted to testis (Tables 1 and 2). It is presumably related to (but distinct from) the steroidogenic acute regulatory gene [58]. More recently, TEX261 was reported to modulate the excitotoxic cell death induced by activation of the N-methyl-D-aspartate receptor - a calcium-permeable ionotropic receptor that has a role in many neurologic disorders [59]). *Tex261*-null mice show defects of the skeleton, immune system, growth/size/body, and adipose tissue (Table 2).

### Tex264

Although *TEX264* expression has been observed in seminiferous duct cells and Leydig cells (according to the Human Protein Atlas), there are no other data on its expression in the testis. In mammalian cells, TEX264 is a major receptor for selective reticulophagy - a process responsible for the specific sequestration of components inside the endoplasmic reticulum alongside the associated ribosomes [60].

### Tex292

*Tex292* is also referred to as *Utp4*. At present, the only data on TEX292 expression in testis corresponds to a report of expression in seminiferous duct cells and Leydig cells. *Tex292* inactivation is embryonic-lethal [61]. The only available data relates to processes or cell types not associated to spermatogenesis or germ cells [61].

## Human *TEX* gene expression and defects

### mRNA expression

Of the 49 *TEX* genes expressed in humans, 13 are testis-specific and 27 show a high expression level in testis, with a mean testis ratio above 0.4 (between 0.411 and 0.998). Of the 9 remaining *TEX* genes, 4 are most strongly expressed in the testis for 4, 3 are more strongly expressed in thyroid or spleen, and 2 are pseudogenes. Data are summarized in Table 5 and detailed in Table 1.

**Table 5:** Human *TEX* gene expression in the testis

	Highest expression in the testis					Highest expression in other tissues
Mean testis ratio	1	1 to 0.8	0.8 to 0.6	0.6 to 0.4	below 0.4	
*TEX* genes	13A, 13B, 13C, 13D, 19, 28, 33, 36-AS1, 36, 37, 51, 55	12, 14, 26-AS1, 34, 35, 38, 39A, 40, 43, 44, 45, 46, 47, 48, 49, 50, 53, 54, 101	11, 15, 22, 26, 29, 39B, 41	21P, 30, 39C	264, 27, 292, 52	261, 39C, 42

### Protein expression

Protein expression data remains scarce. For 14 *TEX* genes, the protein expression pattern is similar to the mRNA expression pattern. A testis-specific protein isoform has been identified for 29 *TEX* genes, and expression data are missing for 14 other genes. An ovary-specific protein isoform has only been identified for *TEX11*. A highly variable testicular location has been reported for 21 *TEX* genes (Table 1), although germinal cell expression (from spermatogonia to spermatids) has been reported in 19 cases. Eight proteins are referenced in the OMIM database (https://omim.org/), and defects in 3 of the coding genes (*TEX11, 14* and *15*) have been linked to phenotypes.

### *TEX* genes, dysregulation of spermatogenesis, and a predisposition to infertility

Thirteen *TEX* genes are now referenced in the OMIM database, and 3 have been linked to a specific phenotype. Here, we report only the data associated with gene defects (Tables 6 and 7).

**Table 6:** Variants of *TEX* genes identified solely in males with azoospermia and/or infertility

***TEX gene***	***Study***	***Nucleotide change***	***Protein change***	***Type of mutation***	***Exon/intron***	***Number of males with the alteration***
*TEX11*	Krausz et al., 2020 [15]	c.84_651del	p.28del189aa	deletion	Exon 4–9	1
	Cannarella et al., 2020 [16]	c.776C → T	p.Thr259Ile	missense mutation	Exon 9	1
		c.2288 T → C	p.Val763Ala	missense mutation	Exon 26	1
	Sha et al., 2018 [17]	c.2653G → T	p.W856C	missense mutation	Exon 29	2 brothers
	Nakamura et al., 2017 [18]	c.511A → G	p.Met171Val	missense mutation	Exon 8	1
	Yatsenko et al., 2015 [19]	c.450C → T	p.A150A	splicing mutation	Exon 7	1
		c.511A → G	p.M171V	missense mutation	Exon 8	1
		c.652del237bp	p.218del79aa	deletion	Exons 10–12	2
		c.792 + 1G → A	p.L264spl d	splicing mutation	Intron 11	1
		c.1837 + 1G → C	p.R612spl d	splicing mutation	Intron 22	1
		c.2092G → A	p.A698T	missense mutation	Exon 25	1
	Yang et al., 2015 [20]	c.-17 T → C	/	intronic alteration	Intron 3	1
		c.-48G → A	/	intronic alteration	Intron 5	1
		c.349 T → A	p.W117R	missense mutation	Exon 6	1
		c.405C → T	/	silent mutation	Exon 6	1
		c.424G → A	p.V142I	missense mutation	Exon 7	1
		c.466A → G	p.M152V	missense mutation	Exon 7	1
		c.515A → G	p.Q172R	missense mutation	Exon 7	1
		c.731C → T	p.T244I	missense mutation	Exon 10	1
		c. + 42C → A	/	intronic alteration	Intron 10	1
		c.-28 T → C	/	intronic alteration	Intron 12	1
		c.-64G → A	/	intronic alteration	Intron 15	1
		c.1258Ins (TT)	1258GATG → TTGGTA	frameshift mutation	Exon 16	1
		c. + 16A → G	/	intronic alteration	Intron 20	1
		c.-1G → A	/	alteration of splicing acceptor site	Intron 21	1
		c.-37A → G	/	intronic alteration	Intron 22	1
		c.-44C → T	/	intronic alteration	Intron 23	1
		c. + 119G → A	/	intronic alteration	Intron 24	1
		c.2243 T → C	p.V748A	missense mutation	Exon 26	1
		c.2319 T → C	/	silent mutation	Exon 27	1
		c.-55A → C	/	intronic alteration	Intron 27	1
		c.-44A → G	/	intronic alteration	Intron 28	1
*TEX14*	Krausz et al., 2020 [15]	c.(554 + 1_555–1)_(3378 + 1_3378–1)del	p.185del941aa	partial deletion	Exon 6–21	1 compound heterozygote
		c.2303_2306del	p.Gln768ArgfsTer31	frameshift deletion	Exon 14	
		c.3454C > T	p.Arg1152Ter	stop gain	Exon 21	1
	Araujo et al., 2019 [21]	c.727C > G	p.Gln243Glu	missense mutation	Exon 7	1 compound heterozygote
		c.4297G > A	p.Glu1433Lys	missense mutation	Exon 31	
	Fakhro et al., 2018 [22]	c.C254A	p.Arg85Leu	missense mutation	/	2 brothers
		c.555-5 T > G	/	splice site mutation	/	1
		/	p.Ser1255fs	frameshift mutation	/	1
	Gershoni et al., 2017 [23]	c.2668-2678del	early stop codon	frameshift deletion	Exon 16	2 brothers
*TEX15*	Cannarella et al., 2020 [16]	c.7118G > A	p.Ser2373Asn	missense mutation	Exon 8	1
	Araujo et al., 2019 [21]	c.7118G > A	p.Ser2373Asn	missense mutation	Exon 8	1 compound heterozygote
		c.9223G > A	p.Gly3075Arg	missense mutation	Exon 10	
	Wang et al., 2018 [24]	c.6934G > A	p.R2312X	nonsense mutation	Exon 1	1
	Colombo et al., 2017 [25]	c.2419A > T	p.Lys807*	nonsense mutation	Exon 8	2: brothers (compound heterozygotes)
		c.3040delT	p.Ser1014Leufs*5	deletion	Exon 8	
	Okutman et al., 2015 [26]	c.2130 T > G	p.Y710*	nonsense mutation	Exon 1	3 brothers

**Table 7:** Polymorphisms in *TEX* genes associated with azoospermia and/or infertility, according to the literature

***SNP ID***	***Study***	***TEX gene***	***Nucleotide change (according to transcript variant 1)***	***Protein change***	***Gnomad (******https://gnomad.broadinstitute.org/******) frequency in the general population***	***Significantly associated with male infertility***	***Population***
rs6525433	Zhang et al., 2015 [60]	*TEX11*	c.389A > G	p.Lys130Arg	0.125	Yes	Chinese
rs4844247			c.1351G > A	p.Glu451Lys	0.103	No	
Association						Yes	
rs323344	Aston et al., 2010 [16]	*TEX15*	c.5158 T > G	p.Leu1720Val	0.148	No	Caucasian
rs323345			c.5081A > G	p.Asn1694Ser	0.168	No	
rs323347	Ruan et al., 2012 [22]		c.1459 T > C	p.Cys487Arg	0.255	Yes	Chinese
rs323346			c.4252A > G	p.Ile1418Val	0.251	Yes	
	Zhang et al., 2010 [60]					No	

#### *TEX11* (OMIM 300311) [15–20, 62, 63]

*TEX11* (on Xq13.1) is currently the most frequently reported gene as being associated with azoospermia [62]. Using an X-chromosome high-resolution microarray, Yatsenko et al. identified the loss of *TEX11* exons 9–11 (607del237bp) in two azoospermic patients with homogeneous or mixed meiotic arrest (47). This in-frame genomic deletion predicted a protein lacking 79 amino acids in the highly conserved sporulation domain SPO22. Additional *TEX11* missense and splicing variants were found in 2.4% of the azoospermic patients but not in any of the 384 men with normal sperm concentrations [19]. Forty variants were subsequently identified by sequencing *TEX11* exons 2 to 30 and the flanking intronic regions in a large cohort of infertile men with nonobstructive azoospermia (*n* = 246) and in fertile controls (*n* = 175), [20]. Twenty-one of these variants were singletons (i.e. each was observed in one individual only), while the remaining 19 were observed in 2 or more infertile men and/or fertile controls. Eighteen were identified solely in patients with azoospermia. The variants include exonic missense mutations, exonic silent mutations, exonic frameshift mutations, and intronic mutations. The researchers concluded that *TEX11* variants were detected with a significantly higher frequency in men with spermatogenic failure than in controls (7.3% versus 1.7%, respectively; *p* = 0.007) [20]. However, the study did not find any differences between pathologic and benign variants. Since then, additional *TEX11* missense variants or deletions have been reported [47,4852,53]; suggesting that this X-linked gene has a major role on azoospermia. Recently, low *TEX11* expression was reported in a man with Sertoli-cell-only (SCO) syndrome [2]. *TEX11* is linked to spermatogenic failure, X-linked, 2 syndrome in the OMIM database (OMIM 309120).

In 2015, Zhang et al. explored the possible association between single nucleotide polymorphisms (SNPs) in *TEX11* and idiopathic male infertility [63]. The homozygous rs6525433 polymorphism genotype was significantly associated with general infertility (odds ratio (OR) = 1.517, 95% confidence interval (CI):1.070–2.150, *p* = 0.019) and oligozoospermia (OR = 1.858, 95% CI: 1.082–3.192, *p* = 0.023) - indicating that the rs6525433 polymorphism has a role in male infertility. The non-synonymous SNP rs6525433 neutralizes the charged amino acid at position 130 of the TEX11 protein (K130A), which might have a negative effect on its structure. No association between the *TEX11* rs4844247 SNP and male infertility was observed. However, carriers of both rs6525433 C and rs4844247 T had an increased risk of infertility (95% CI: 1.042–2.542) [63].

#### *TEX12* (OMIM 605791)

As in mice, human *TEX12* is reportedly essential for the synaptonemal complex [33]. Even though low TEX12 expression has been reported in a patient with SCO syndrome [2, 32], further studies are required to confirm the link between *TEX12* variants and defective spermatogenesis.

#### *TEX14* (OMIM 605792) [15, 21–23, 64]

As in mice, human TEX14 is essential for forming stable intercellular bridges in germ cells [36]. To date, few *TEX14* genetic variants have been linked to spermatogenesis failure. The first (a 10-bp deletion) variant was identified in 2017 in two infertile brothers with nonobstructive azoospermia from a consanguineous Iraqi Jewish family [23]. The variant leads to a frameshift in the *TEX14* coding region and thus results in an early stop codon and a truncated protein. Other deleterious variants have been associated with infertility, maturation arrest, and SCO phenotypes [22, 64]. Taken as a whole, these data suggests that alterations in *TEX14* gene has a major impact on the onset of azoospermia. *TEX14* is now also listed in the OMIM database as being linked to spermatogenic failure 23 syndrome (OMIM 617707). Furthermore, low TEX14 expression has been reported in a patient with the SCO syndrome [2].

#### *TEX15* (OMIM 605795) [2, 26, 37, 63, 65, 66]

The first nonsense mutation (leading to a premature stop codon) in the *TEX15* locus was identified by exome sequencing in a consanguineous Turkish family [26]. The mutation co-segregated with the infertility phenotype; two brothers with nonobstructive azoospermia and an oligozoospermic sibling were homozygous for the mutation. These males presented a drastically reduced testicular size (by more than 50%) and maturation arrest at the primary spermatocyte stage [26]. Other non-consanguineous siblings with nonobstructive azoospermia and a low testicular volume have been found to be compound heterozygotes for deleterious *TEX15* variants [25]. To date, few damaging variants have been identified (Table 4). *TEX15* has now been linked to spermatogenic failure 25 syndrome in the OMIM database (OMIM 617960). Furthermore, low TEX15 expression has been reported in a man with SCO syndrome [2].

Various studies have assessed the association between *TEX15* polymorphisms and male infertility. One study did not find an no association [65]. In 2015, Ruan et al. analyzed the distribution of SNPs of the *TEX15* gene within a male Chinese Han population. The researchers reported that two genetic variants (rs323346 and rs323347) in *TEX15* gene conferred susceptibility to spermatogenic failure [66]. However, this finding was not confirmed by Zhang et al. for rs323346 [63].

#### *TEX101* (OMIM 612665)

In 2013, TEX101 was first suggested as a biomarker for the differential diagnosis of azoospermia [57]. Schiza et al. used an ELISA assay to (i) evaluate the seminal plasma level of TEX101 and the success of vasectomy, (ii) stratify forms of azoospermia, and (iii) better select patients for sperm retrieval. The same group used differential proteomic profiling to evaluate the impact of the common *TEX101* missense variant rs35033974 in infertile men with various etiologies. They reported that 8 cell surface proteins and 9 testis-specific secreted proteins were significantly down-regulated in four patients who were homozygous for rs35033974. The researchers have also found that the seminal plasma level of TEX101 in heterozygous males was five times lower (*p* = 0.0005) that in controls [57]. Schiza et al. concluded that the *TEX101* rs35033974 variant could then be taken into account in diagnosis of infertility.

## Conclusion

**Fig. 2:**
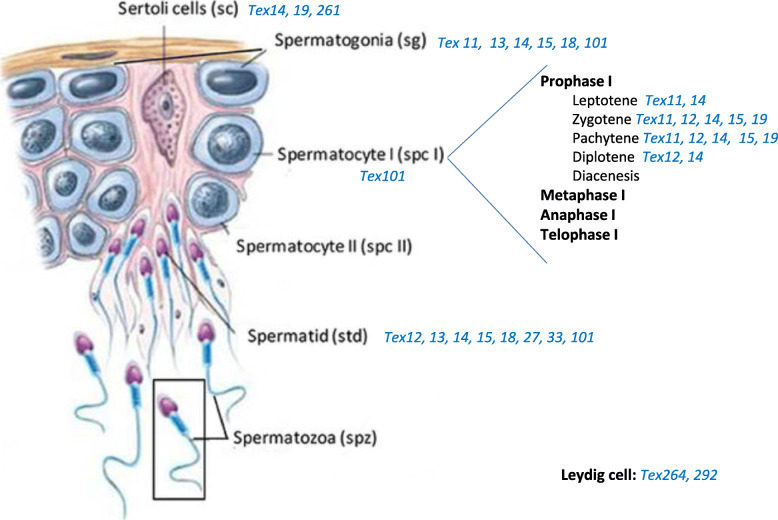
Implication of TEX genes during spermatogenesis (Adapted from [67])

As expected, TEX genes appear to have a major role in reproduction in general and in spermatogenesis in particular. *As the only common feature of TEX genes is their expression in the testis, the genes are involved in many different pathways and functions (*Fig. 2*) in testis cells, germ cells (from spermatogonia to spermatids), Sertoli cells, and Leydig cells.* This is true not only in humans but also in all mammals such as the mouse and the rat. In the future, cumulative data on the human TEX genes’ physiological functions and pathophysiological dysfunctions should become available Furthermore, further studies of the functional effects of natural knockouts or knockdowns in humans are necessary for defining the list of essential and nonessential testis-specific genes and proteins and thus advancing the biology of human reproduction.

## Data Availability

not applicable.

## Abbreviations

CAPZA 3: Capping actin protein of muscle Z-line subunit alpha 3
CATSPERZ: CATion channel, SPERm-associated, auxillary subunit Zeta
cDNA: Complementary DNA
cM: Centimorgan
DMC1: DNA Meiotic reCombinase 1
DSB: Double-strand breaks
ELISA: Enzyme-linked Immunosorbent Assay
FANTOM: Functional annotation of the mammalian genome
HTNS: High in testis but not specific
KO: Knock out
MILI: Miwi-like
MIWI2: (Another PIWI family member)
MT: Majority testis
NA: Non available
NP: No predominance
OMIM: Online mendelian inheritance in man
PIWI: P-element induced wimpy testis
RAD51: RAD51 Recombinase
SCO: Sertoli-cell-only
SIX6OS1: Six6 opposite strand transcript 1
SMC3: Structural maintenance of chromosomes protein 3
SNP: Single nucleotide polymorphisms
SSH: Suppression subtractive hybridization
Sycp2: Synaptonemal complex protein 2
SYCP1–3: Synaptonemal complex protein 1–3
SYCE1–3: Synaptonemal complex central element protein 1–3
Tctex: T-complex testis-expressed
Tex: Testis expressed
TS: Testis specific
Utp4: U3 small nucleolar RNA-associated protein 4 homolog
Zfand3: Zinc finger AN1 domain-containing protein 3
